# Automatic Segmentation of Osteonal Microstructure in Human Cortical Bone Using Deep Learning: A Proof of Concept

**DOI:** 10.3390/biology12040619

**Published:** 2023-04-19

**Authors:** Alina Littek, Stephen J. McKenna, Wei Xiong Chiam, Elena F. Kranioti, Emanuele Trucco, Julieta G. García-Donas

**Affiliations:** 1 Computer Vision and Image Processing Group, School of Science and Engineering, University of Dundee, Dundee DD1 4HN, UK; alina.littek@gmail.com (A.L.); 2478626@dundee.ac.uk (W.X.C.); e.trucco@dundee.ac.uk (E.T.); 2 Forensic Medicine Unit, Department of Forensic Sciences, School of Medicine, University of Crete, 70013 Heraklion, Greece; ekranioti@uoc.gr; 3 Centre for Anatomy and Human Identification, School of Science and Engineering, University of Dundee, Dundee DD1 5EH, UK

**Keywords:** deep learning, semantic segmentation, cortical bone microstructure, osteons, identification

## Abstract

**Simple Summary:**

Human cortical bone microstructure assessment is used in biological and forensic anthropology for different purposes. For example, studies have investigated the relationship between the microstructural features and age, while others have examined bone microstructure for identification of animal and human bone. The present research is a pilot study investigating the possibility of automatic analysis of human bone microstructure microphotographs through the application of deep learning. The aim of this study is to explore the feasibility of identification of intact and fragmentary osteons in human cortical bone. Our results demonstrate the potential of deep learning for differentiation of osteonal structures, although a larger dataset and further refinement of the model is required in the future to confirm our preliminary results and provide a more accurate identification of osteonal structures.

**Abstract:**

Cortical bone microstructure assessment in biological and forensic anthropology can assist with the estimation of age-at-death and animal-human differentiation, for example. Osteonal structures within cortical bone are the key feature under analysis, with osteon frequency and metric parameters providing crucial information for the assessment. Currently, the histomorphological assessment consists of a time-consuming manual process for which specific training is required. Our work investigates the feasibility of automatic analysis of human bone microstructure images through the application of deep learning. In this paper, we use a U-Net architecture to address the semantic segmentation of such images into three classes: intact osteons, fragmentary osteons, and background. Data augmentation was used to avoid overfitting. We evaluated our fully automatic approach using a sample of 99 microphotographs. The contours of intact and fragmentary osteons were traced manually to provide ground truth. The Dice coefficients were 0.73 for intact osteons, 0.38 for fragmented osteons, and 0.81 for background, giving an average of 0.64. The Dice coefficient of the binary classification osteon-background was 0.82. Although further refinement of the initial model and tests with larger datasets are needed, this study provides, to the best of our knowledge, the first proof of concept for the use of computer vision and deep learning for differentiating both intact and fragmentary osteons in human cortical bone. This approach has the potential to widen and facilitate the use of histomorphological assessment in the biological and forensic anthropology communities.

## 1. Introduction

In biological and forensic anthropological analysis, the macroscopic observation of bone allows the expert to understand key information about an individual, such as population affinity, sex, age and stature, which correspond to the four main pieces of information gathered for the construction of the biological profile [1]. When the remains are highly fragmented or affected taphonomically, the assessment of cortical bone microstructure is the last resource before opting for biochemical processes [2]. From the bone tissue types, the outer highly mineralized layer of tissue provides resistance to further taphonomic alterations [3]. Thus, cortical bone histomorphometry can assist the disciplines offering insights into age-at-death, differentiation between animals and humans, and the identification of pathological conditions [4,5,6]. 

Within cortical bone, osteons are the main feature assessed for most anthropological analysis [7,8,9]. Osteonal structures formed by basic multicellular units represent the traces of cyclic remodeling events [10,11]. Methods are therefore developed using the osteon as the key parameter, with measurements taken in relation to their frequency per bone area, size and shape [7,9,12]. Qualitative analysis is commonly performed with osteons being differentiated as intact or fragmentary depending on different degrees of resorption [9]. Other osteons are differentiated based on their morphological characteristics, as for example drifting or double zonal osteons, providing information about metabolic or biomechanical demands [13,14]. 

Technically, bone cross-sections are processed for histomorphometric assessment manually or with special equipment by grinding and polishing the bone surface until the microscopic features can be observed with enough detail for the expert to perform the analysis [15,16]. As for the data collection approach, the manual counting or manual measuring of osteonal structures can be conducted using a standard research microscope and image analysis software such as ImageJ [17,18]. Regardless of population differences, the average amount of intact and fragmentary osteons for the rib cross-section of a 60-year old individual is approximately 300, with the clavicle cross-section having 1220 at the age of 40 [19,20]. The data collection protocol for cortical bone histomorphometry entails a time-consuming process requiring specialized training as well as experience to ensure accurate feature identification [21,22]. 

Automated data collection processes have emerged in recent years, including software tools and machine learning-based methods [23,24,25]. Approaches such as neural networks or random forests have been applied for population affinity, sex and age estimation [24,26]. The only osteon segmentation system using deep learning known to us was designed for osteo-histological segmentation of non-avian theropod dinosaurs [27]. Images are substantially different (e.g., sparse, mostly isolated target structures; largely uniform background), making that system arguably unsuitable for images of human osteons like the ones used in our study. Although further validation is required to ensure reliable outcomes [28,29], automated approaches have shown promising results as a means to enable more sophisticated statistical analysis, data interpretation and decision making [30].

Our study is part of a research program exploring the potential of machine learning for assessing cortical bone microstructure. A proof-of-concept system for segmenting osteons is reported, along with tests on a pilot data set with ground-truth annotations. To the best of our knowledge, this paper presents the first attempt to automate the histomorphometric anthropological assessment of intact and fragmentary osteons in human cortical bone using artificial intelligence techniques.

## 2. Materials and Methods

### 2.1. Dataset

The sample under study consists of microphotographs obtained from rib thin sections that were collected from a previous study and includes the mid-segment of standard ribs from individuals with a mean age of 60 (SD = 17.89) [19].

The histological slides were prepared using standardized procedures [19,31]. High quality microphotographs were captured at 100x magnification under semi-polarized settings using a Leica DM 750P research microscope fitted with a Leica MC 170 HD camera and Leica Application Suite V4 software (Leica Microsystems, UK). The dimensions in pixels were 2592 × 1944 and 1600 × 1200. Although only semi-polarized microphotographs were used for the machine learning analysis, transmitted light microphotographs were also used for further confirmation of structure identification on very crowded cortices (e.g., high frequency of osteonal structures on oldest age individuals). 

**Figure 1:**
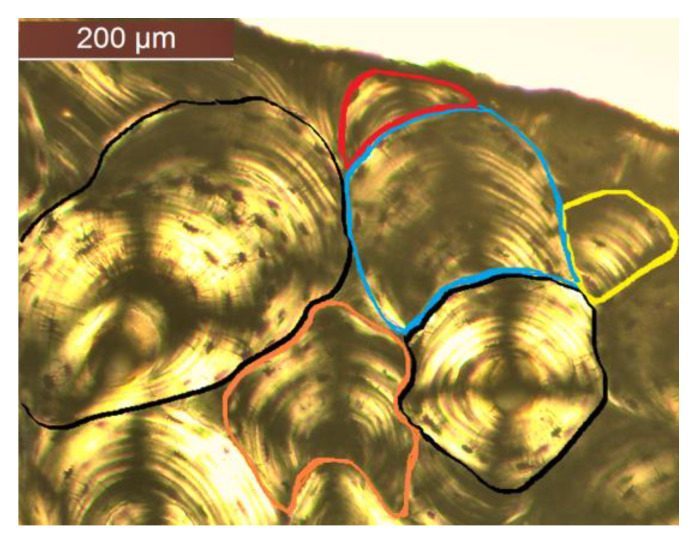
Examples of intact osteons (black outlines) and fragmentary osteons (colored outlines) identified following the criteria for this study.

Only secondary osteons were assessed, as these are the ones usually quantified for the anthropological assessment [7,8,32,33,34]. A protocol for secondary osteon identification was created for consistency purposes. The two types of osteons most commonly assessed in previous studies were differentiated: intact osteons and fragmentary osteons [7,9,19,32,35]. The definitions followed for identification were those from Stout and Paine [9]. Intact osteons were classified as such if the Haversian canal showed at least 90% of its perimeter with no sign of resorption, while fragmentary osteons were identified as having more than 10% of their canals showing signs of remodeling (Figure 1). For those osteons that were cut by the frame of the microphotograph, both the Haversian canal and concentric lamellae and osteocytes arrangement were used for reference, with the canal visibility criteria being used (e.g., a fragmentary osteon was identified if the canal perimeter was cut by more than 10%). Drifting osteonal structures were classified as intact or fragmentary based on the aforementioned criteria and following guidelines proposed by Robling and Stout [14]. Type II and double zonal osteons were identified following descriptions and guidance from previous studies [36,37].

**Figure 2:**
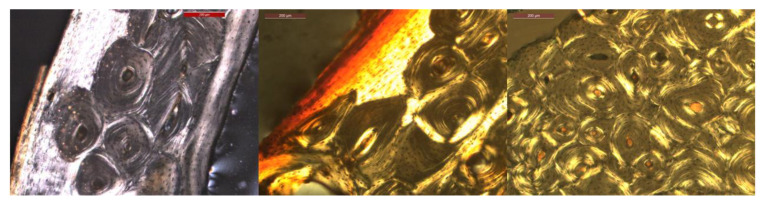
Three images illustrating variations in osteon size, color and shape.

Significant variations in color, shape, and portion of bone are present, making automatic segmentation challenging. In addition, some of the images show colored markings on the bone that were added during the preparation for the thin sections. An illustration of variations in size, color and shape is shown in Figure 2.

### 2.2. Manual Image Annotation 

**Figure 3:**
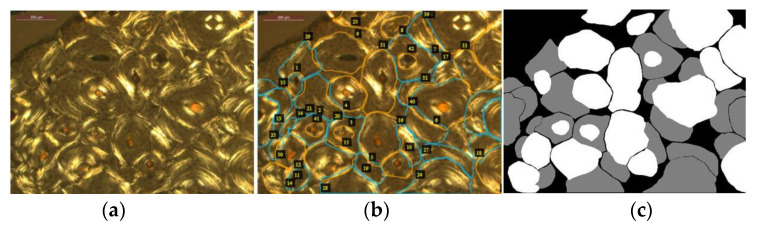
An example of mask generation: (**a**) original microphotograph; (**b**) annotations drawn on the original image (**a**) with examples of classes ‘intact’ and ’fragment’ marked in orange and blue, respectively; all other pixels are considered background; (**c**) the corresponding mask image.

Intact and fragmentary osteons were manually annotated on 99 microphotographs using the VGG Image Annotator (www.robots.ox.ac.uk/~vgg/software/via/ (accessed 30 January 2022)), a user-friendly and lightweight software application appropriate for tracing image regions. Annotations were performed by a single observer with extensive experience in bone histomorphometry (JGGD). Intact and fragmentary osteons were identified according to the protocol described above. The remaining image regions were considered background. Contours were approximated by polygons with no restrictions on the number of sides; the *polyline* tool was chosen to select points delimitating the region of the osteons. A total of 1415 individual osteons were identified and annotated, consisting of 773 intact osteons and 642 fragmentary osteons. Annotations of overlapping structures were performed in a manner dependent on their classes; the boundary was drawn to overlap if an intact osteon was located on top of, or overlapping with, a fragmentary osteon. When two instances of the same class overlapped (i.e., intact over intact or fragmentary over fragmentary), then a small space was kept between objects. Masks were generated from the annotations in which each pixel was associated with an integer number representing one of the three classes (intact osteon, fragment, background). An example of annotated regions is shown in Figure 3. 

### 2.3. Deep Learning Model: Semantic Segmentation

Given a dataset of training pairs, in which each pair consists of an image and its corresponding segmentation mask (such as in Figure 3a,c), a neural network can be trained to map images to segmentation masks. Given sufficient training data these networks can generalize, producing sufficiently high-quality segmentations of previously unseen images from the same domain as the training data. Training uses an optimization algorithm that iteratively adjusts internal parameters in the neural network so as to reduce its loss (some measure of error) on the training data. Training deep neural networks with many layers of ‘neurons’ to classify or segment images is now common practice in computer vision and biomedical image analysis, achieving state of the art results on many tasks [38].

U-shaped neural network architectures have proven effective for image segmentation [39]. These architectures consist of an encoder part followed by a decoder part. The encoder part can be thought of as mapping the input image to multiple feature maps that together comprise a representation of the input image. The decoder part then maps these feature maps to a segmentation map, which is the same size as the input image. Skip connections which directly connect layers of neurons in the encoder to layers of neurons in the decoder help the network produce segmentation maps with finely detailed high-resolution information. 

The deep learning network we adopted was a modified U-Net [39] implemented in Python using Keras. All images were resized to 512 × 512 pixels using bilinear interpolation and converted to greyscale for further processing. U-Net layer sizes were set to accommodate the 512 × 512 input images. Given the relatively small size of our dataset, data augmentation was used to reduce overfitting. Data augmentation is common practice when deep learning is applied to image analysis tasks; by applying transformations to the training images and their segmentation masks, the amount of training data is synthetically increased, helping to promote generalization. Specifically, data were augmented during training by randomly applying horizontal and vertical flipping, zooming (−20% to +20%), 90-degree rotations, and shearing (with angle 0.2 radians) to the input images. 

Dropout (dropping different sets of neurons randomly) was also used to reduce overfitting during training. The dropout rate was set to 0.3. Zero-padding was used at the image boundary to make the size of the output the same as that of the input image. The output layer used softmax activation to generate a probability value per pixel for each of the 3 classes. In total, the network had 1,940,851 trainable parameters. Training was performed using the widely used Adam stochastic gradient-based optimizer [40] with a weighted categorical cross-entropy loss. The batch size was 2 and early stopping with a patience parameter of 25 iterations was used. 

The output from the final layer of the trained network can be treated as providing a probability distribution over the three class labels for each pixel in the image. These probabilities can be used for further processing. Alternatively, a labelling of the image (i.e., an automatic image annotation) can be obtained by assigning each pixel the label with highest probability. This labelling can be compared with the manually obtained expert annotation (the ‘ground truth’) to quantify segmentation performance. 

## 3. Results

### 3.1. Qualitative Results

**Figure 4:**
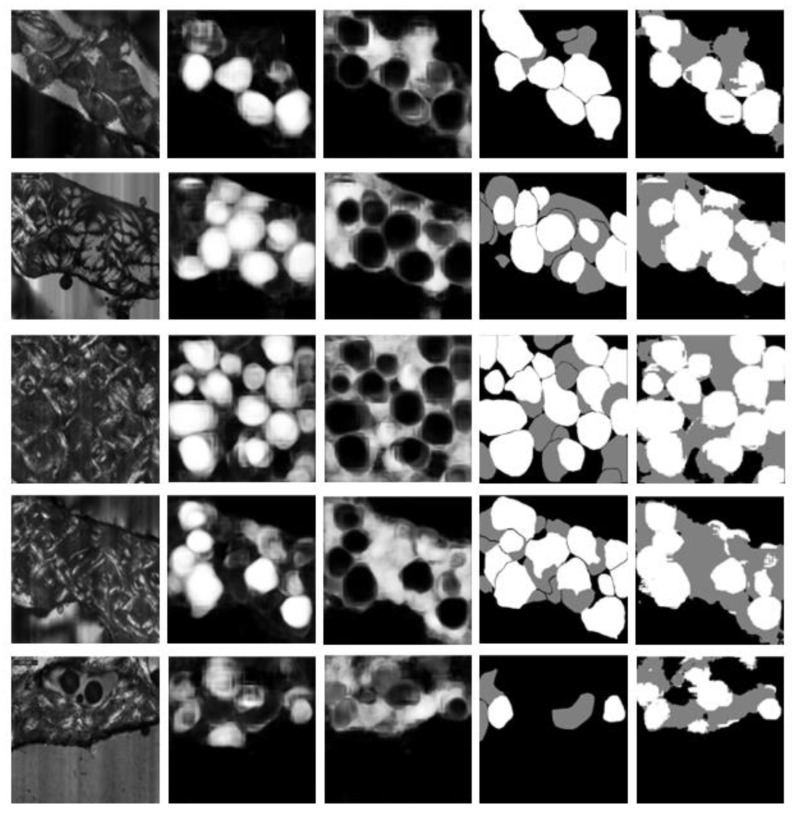
In each row, left to right: original image, predicted probability map for intact osteons, predicted probability map for fragmented osteons, manual annotation (ground truth), and automatic segmentation (after assigning each pixel to the class with highest predicted probability).

Figure 4 shows example test results for images presenting different levels of difficulty. Counting from the top, rows 1, 2 and 3 show examples of good segmentations: most osteons are captured well with only minor errors. Rows 4 and 5 show examples of less successful segmentations. In Row 4, there are substantial false negatives for intact osteons, although the background is captured well. Row 5 presents a texture that is poorly represented in the training set, leading to extensive false positives for both intact and fragmented osteons.

### 3.2. Quantitative Results

Table 1 is the normalized confusion matrix computed over all 99 images when used as test images (in the context of 10-fold cross-validation). This matrix shows how the manual and automatic pixel class assignments were jointly distributed. Quantitative evaluation metrics were computed from this matrix. The proportions of pixels in each class according to the manual annotations (obtained by summing the rows of the confusion matrix) were 54.7% background, 13.5% fragment, and 31.8% intact osteon. The corresponding proportions predicted automatically (obtained by summing the columns of the confusion matrix) were 46%, 21%, and 33%, respectively. Intact osteon pixels were identified with a sensitivity (true positive rate) of 74% and a specificity (true negative rate) of 86%.

The Dice coefficient is a commonly used metric when evaluating semantic segmentation. For a single class, it is computed as

(1) $$\frac{2TP}{2TP+FP+FN}$$

where *TP*, *FP*, and *FN* are the numbers of true positive, false positive, and false negative pixels, respectively, considering instances of the class in question to be positive. Dice coefficients obtained were 0.73 for intact osteons, 0.38 for fragmented osteons, and 0.81 for background, giving an overall (average) Dice coefficient of 0.64. 

Considering the two-class problem discriminating between osteonal structure and background (i.e., collapsing the intact and fragment classes into a single class), the Dice coefficient is 0.82, and osteonal pixels were identified with a sensitivity of 88% and a specificity of 74%.

## 4. Discussion

Bone histomorphometry is a valuable tool for biological and forensic anthropology. Biological anthropological research has been performed exploring robusticity and its potential correlation with microscopic parameters [41] understanding intra- and inter-population variation regarding osteon dimensions [42], and intra-cortical differences between mammals for species identification [43], among others. Regarding forensic anthropology applications, multiple studies have been produced investigating different topics such as development and validation of age-at-death methods, human versus human identification, as well as the effect of heating on cortical bone microstructure and its implications for forensic identification [35,44,45,46]. Based on the wide range of topics covered, the crucial contribution that cortical bone histomorphometry makes to the field is evident.

Histological analysis of bone requires the preparation of thin-sections, and different protocols have been proposed depending on the equipment available, nature of the research question, and state of preservation of the material [15,31,47,48]. Thus, efforts have been made to provide different options for bone sample processing. 

Regarding data collection, one of the drawbacks of the microscopic approach relates to the qualitative and quantitative assessment of the microstructural features, which is a time-consuming process. Moreover, issues have been highlighted regarding difficulties in the identification and quantification of some histomorphometric parameters, suggesting that the assessment should be performed by a trained researcher [22,49]. For example, studies have reported difficulties with the identification of fragmentary osteons [22,50], with this parameter being crucial for representing the remodeling rates, and thus, essential for accurate age estimation [9]. In addition, the nature of the sample will also have an impact on the observation of the microscopic features, with human bone from advanced age individuals posing a challenge. With age, the number of osteonal structures increases while osteon area and cortical area decrease producing what is known as the packing effect, resulting in the crowding of osteons within the sampling area being assessed [51]. As age advances, the difficulty of their identification becomes apparent.

In view of exploring a possible solution to the difficulties of cortical bone histomorphometric data collection, the present study aimed to test the application of machine learning for the automatic identification of intact and fragmentary osteons. A semantic segmentation protocol was used on 99 microphotographs collected from human ribs for which annotations were made for both types of osteons. The results provided by the U-Net developed here produced a higher Dice coefficient for intact osteons than for fragmentary osteons (0.73 and 0.38, respectively). Although intact osteons also differ in shape and size, fragmentary osteons present much more variation as they are the results of an intact osteon appearing as a new remodeling event and overlapping with the existing structures (either intact or fragmentary osteons). Based on this, fragmentary osteons are highly inconsistent producing structures accounting for a wide range of shapes and dimensions (refer to Figure 1). 

Considering the results obtained for osteonal structures (combining intact and fragmentary osteons), the Dice coefficient is 0.82. Other studies have been published regarding cortical microstructural identification. For example, Qin et al. [27] recently developed a deep convolutional network model to identify primary and secondary osteons through vascular canals and circular lamellar bone on paleontological specimens. The results from that study provided a Dice coefficient ranging from 0.74–0.75 for secondary osteons identification. As seen throughout the fossil microphotographs [27], the microstructures are well spaced when compared to the human samples used in our study, suggesting that the segmentation problem would be easier to solve. 

Our study suggests that the U-Net shows potential for human osteonal structure identification, although further refinement is needed to obtain more accurate identification of both intact and fragmentary osteons. This pilot aimed to explore the general suitability of deep learning methods for human cortical osteon segmentation. Hence, only one deep learning model (architecture) was used. Given a larger set of annotated images, it would be worth testing the performance of different models. Additionally, further research could incorporate other bones such as the femur, which is also frequently used for anthropological analysis [52,53]. Moreover, the inclusion of microphotographs with different levels of resolution could be tested to see if microphotograph quality has an impact on the results. Ideally, further research will focus not only on refining the semantic segmentation of osteonal structures but will also explore the possibility of counting and measuring those structures, via instance segmentation, since osteon frequency and osteon size are commonly assessed in histomorphometric methods [5,9,19]. In the future, an automatic osteon identification tool available to researchers and practitioners would imply that data collection would become less time-consuming, less subjective, and more accurate. Large datasets for research purposes could be assessed by an online tool saving time for the researcher. Additionally, it could also offer the possibility of assessing the whole cross-section instead of considering a sampling strategy. Moreover, the availability of the software could enable the incorporation of bone histomorphometry in forensic anthropology as a routine in medicolegal settings. 

## 5. Conclusions

This study aimed to explore the potential of artificial intelligence for the automatic identification of the key parameter in human cortical bone, the osteon. Considering the very modest number of images available for training a deep learning network, our pilot system achieves good segmentation. Further work using a larger number of images could potentially increase the accuracy reported here. A tool for automatic identification of intact and fragmentary osteonal structures in human cortical bone will assist the biological and forensic anthropology communities in the application of histomorphometry in their routine assessments. 

## Figures and Tables

**Table 1:** Confusion matrix obtained from 10-fold cross-validation.

	Predicted Label		
	Background	Fragment	Intact
Background	0.406	0.090	0.050
Fragment	0.025	0.066	0.044
Intact	0.028	0.054	0.236

## Data Availability

The computing data presented in this study can made available on request from the corresponding author.
